# Leveraging ongoing research to evaluate the health impacts of South Africa's salt reduction strategy: a prospective nested cohort within the WHO-SAGE multicountry, longitudinal study

**DOI:** 10.1136/bmjopen-2016-013316

**Published:** 2016-11-30

**Authors:** Karen Charlton, Lisa J Ware, Elias Menyanu, Richard Berko Biritwum, Nirmala Naidoo, Chiné Pieterse, Savathree (Lorna) Madurai, Jeannine Baumgartner, George A Asare, Elizabeth Thiele, Aletta E Schutte, Paul Kowal

**Affiliations:** 1 School of Medicine, University of Wollongong, Wollongong, New South Wales, Australia; 2 Hypertension in Africa Research Team (HART), North-West University, Potchefstroom, South Africa; 3 Department of Community Health, University of Ghana, Legon, Ghana; 4 World Health Organization (WHO), Geneva, Switzerland; 5 Global Clinical & Viral Laboratories, Durban, South Africa; 6 Centre of Excellence for Nutrition, North-West University, Potchefstroom, South Africa; 7 Chemical Pathology Unit, Department of Medical Laboratory Sciences, University of Ghana, Legon, Ghana; 8 Vassar College, Poughkeepsie, New York, USA; 9 MRC Research Unit for Hypertension and Cardiovascular Disease, North-West University, Potchefstroom, South Africa; 10 University of Newcastle Research Centre for Generational Health and Ageing, Newcastle, New South Wales, Australia

**Keywords:** Salt reduction, Sub-Saharan Africa

## Abstract

**Introduction:**

Attempting to curb the rising epidemic of hypertension, South Africa implemented legislation in June 2016 mandating maximum sodium levels in a range of manufactured foods that contribute significantly to population salt intake. This natural experiment, comparing two African countries with and without salt legislation, will provide timely information on the impact of legislative approaches addressing the food supply to improve blood pressure in African populations. This article outlines the design of this ongoing prospective nested cohort study.

**Methods and analysis:**

Baseline sodium intake was assessed in a nested cohort of the WHO Study on global AGEing and adult health (WHO-SAGE) wave 2 (2014–2015), a multinational longitudinal study on the health and well-being of adults and the ageing process. The South African cohort consisted of randomly selected households (n=4030) across the country. Spot and 24-hour urine samples are collected in a random subsample (n=1200) and sodium, potassium, creatinine and iodine analysed. Salt behaviour and sociodemographic data are captured using face-to-face interviews, alongside blood pressure and anthropometric measures. Ghana, the selected control country with no formal salt policy, provided a nested subsample (n=1200) contributing spot and 24-hour urine samples from the SAGE Ghana cohort (n=5000). Follow-up interviews and urine collection (wave 3) in both countries will take place in 2017 (postlegislation) to assess change in population-level sodium intake and blood pressure.

**Ethics and dissemination:**

SAGE was approved by the WHO Ethics Review Committee (reference number RPC149) with local approval from the North-West University Human Research Ethics Committee and University of the Witwatersrand Human Research Ethics Committee (South Africa), and University of Ghana Medical School Ethics and Protocol Review Committee (Ghana). The results of the study will be published in peer-reviewed international journals, presented at national and international conferences, and summarised as research and policy briefs.

**Strengths and limitations of this study**

- Rigorous random selection procedure based on a sample designed to be nationally representative.
- Ongoing data collection within the WHO Study on global AGEing and adult health (WHO-SAGE) cohort to capture salt intake and disease.
- We were unable to identify the specific foods contributing to salt intake in wave 2.
- Results may be less representative for the smaller sample of adults aged 18–49 years.
- Nationwide implementation of salt regulations precludes inclusion of a local control group.

## Current study status

Data collection for wave 2 (baseline for the nested salt substudy) has been completed, while data collection for wave 3 (first follow-up of the nested salt substudy) will take place in 2017 and is estimated to be complete by the end of 2017. Further waves of data collection are planned as part of the multicountry, longitudinal WHO Study on global AGEing and adult health (WHO-SAGE).

## Introduction

Many adults consume more salt daily than is recommended, contributing to the global epidemic of hypertension and cardiovascular disease (CVD).^1^ In low-income and middle-income countries (LMIC), a disproportionately rapid increase in hypertension is occurring without commensurate strategies to halt this growth or mitigate the impact on CVDs and death.^2^ The scale of the problem in South Africa is evident: among six LMICs in one study, South Africa was shown to have the highest prevalence of hypertension (78%) in adults aged 50 years and older.^3^ ^4^ Public health strategies are being employed to reduce levels of hypertension, including over 30 countries using legislative changes as part of their salt reduction strategy to meet the WHO and World Health Assembly salt target of 30% reduction in population salt/sodium intake by 2025.^5^ ^6^

South African researchers found that non-discretionary salt intake (salt already in processed foods) is estimated to contribute around 60% of the overall daily salt intake, primarily from bread and meat products.^7^ As a result of this research, in March 2013 South Africa was the first country to legislate for mandatory reformulation of a range of foods,^8^ setting maximum sodium levels (mg per 100 g) in targeted processed foods (bread 400 mg; breakfast cereal 500 mg; butter and margarine 550 mg; potato crisps 650 mg; salty snacks 800 mg; raw sausage 800 mg; processed meat 850–950 mg; instant noodle mix 1500 mg; dry soup powder 5500 mg; and stock concentrate 18 000 mg), all identified as contributing significantly to sodium intake in the South African population.^7^ ^9^ ^10^ The first phase of sodium legislation was implemented in June 2016, with further reductions required in sodium levels across food categories by June 2019.^11^ The legislation is predicted to decrease nationwide salt intake by 0.85 g/day;^12^ reduce annual CVD deaths by 11%; save the government US$51.25 million/year and prevent 2000 cases of poverty annually as a result of saving households more than US$4 million/year in out-of-pocket medical expenses.^13^

A number of collateral issues arise regarding salt intake, such as salt being the main source of iodine fortification, so that successful campaigns to reduce salt intake would have the potential to result in reduced iodine intake.^14^ Another consideration is that dietary sodium-to-potassium ratio may predict CVD mortality better than sodium intake alone.^15^ Additionally, debate continues on the efficacy of legislative versus non-legislative approaches to population salt reduction,^8^ ^16^ ^17^ including the mechanisms to monitor and enforce such legislation.^18^ From a public health perspective, the major challenge is to evaluate the effects on population health. This paper discusses one effort undertaken to assess a number of health impacts following the sodium legislation in South Africa in comparison to Ghana, an African country with no sodium legislation, through implementation of a large health study in each country.

## Methods and analysis

### Objectives of the research

The primary aim of this study is to evaluate the impact of the sodium legislation on population sodium intake and blood pressure (BP). Secondary objectives are to assess: (1) the relationship between sodium and potassium levels and BP prelegislation and postlegislation; (2) the impact of the sodium legislation on population iodine intake; and (3) the use of spot urine samples as a proxy for 24-hour urine sample collection to measure population salt excretion in a nested biochemical analysis.

### Theory of change

**Figure 1:**
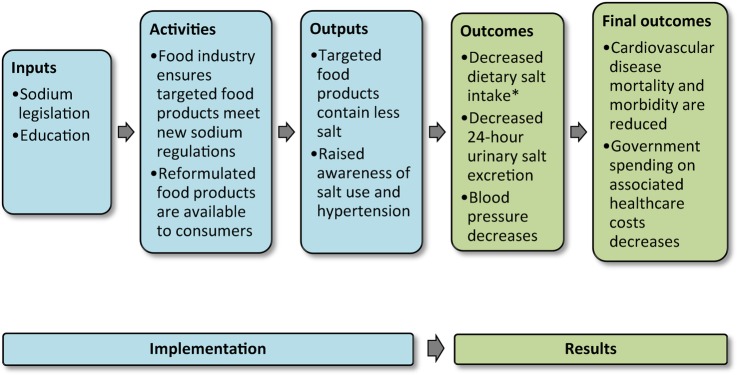
Results Chain model for the SAGE nested salt substudy. Note: adapted from Gertler *et al*.^19^ *Discretionary and non-discretionary salt use.

In order to evaluate the impact of policy on the desired outcome, it is critical to appreciate the steps required between policy implementation and change in the outcome. Gertler *et al*^19^ refer to this as the theory of change (how an intervention is supposed to deliver the required results) and propose various models that can be used for this purpose. One of these models, ‘the Results Chain’, has been adapted for our purposes (figure 1) to explore the causal logic underlying the expectation that sodium legislation will impact BP and CVD. The model shows the inputs (legislation and education), activities (food industry formulation changes; also with monitoring of educational/media activities), outputs (change in sodium levels in foods; changes in awareness about dietary salt and hypertension from any educational activities) and outcomes (reduced dietary intake and urinary excretion of salt, reductions in BP, and finally, reductions in CVD mortality, morbidity and associated healthcare spending).

Inputs, activities and outputs collectively describe the implementation of the legislation and activities planned by the South African National Department of Health (NDoH) and others. These are the subject of monitoring and process evaluation. The outcomes (results) are the subject of impact evaluation and the main focus of this study. However, the impact evaluation needs to be informed by an assessment of the implementation in order to correctly interpret the results.^19^ For example, without knowing whether the targeted food products comply with the legislation, it would be difficult to attribute change in salt intake to the sodium legislation. As such, a multidisciplinary group including the NDoH will be undertaking these assessments, with contributions from the SAGE collaboration. Gertler *et al*^19^ also suggest that development of the Results Chain model promotes exploration of these assumptions and risks within the proposed causal logic. The assumptions and risks are further explored within the discussion.

### Ghana as a comparison group

Gertler *et al*^19^ also recommend including a comparison group. Ideally, this would be a South African group matched on all characteristics and exposures but not affected by the legislation, with data collected at the same time as those who would be affected by the legislation. However, since the legislation is in force across the whole country, this is not possible. Neighbouring countries such as Lesotho, Swaziland, Botswana, etc, would also not be good candidates for a comparison group as many of the South African food manufacturers export goods to these countries under the South African Development Community (SADC) Regional Free Trade agreement (2008). South Africa is the major source of processed snack food in the SADC region,^20^ providing around 80% of the processed food in Zambia, Namibia and Botswana.^21^ These countries then would most likely be affected by the spillover of the South African sodium legislation. SADC countries form over 80% of South Africa's export market, with exports to the rest of Africa consisting mainly of vehicles and machinery.^22^ As such, Ghana, a non-SADC country and the only other African country to implement WHO-SAGE, was selected as the comparator country. Ghana's adult population is also afflicted by a high prevalence of hypertension (up to 48% of adults generally^23^ and 54% of adults 50+ years of age), increasingly poor risk factor profiles (diet, obesity, physical activity), and poor rates of hypertension awareness and control.^3^ In terms of salt intake, the Ghana Health Service has focused primarily on eradication of iodine deficiency through salt iodisation and education.^24^ At the same time, efforts are underway to boost the salt production industry in Ghana as a method of economic development.^25^ ^26^ Salt intakes appear comparable between Ghana and South Africa with studies suggesting that both countries have intakes between 2.3 and 5.5 g sodium/day (equivalent to 5.8–13.8 g salt/day), and higher intakes in urban compared with rural populations.^27^ While there have been some efforts to lower salt intake in community interventions,^28^ until now there appears little evidence to suggest that either mandatory or voluntary sodium targets exist between government and the food industry in Ghana to promote a reduction in population salt intake. However, as in South Africa, it will be critical to monitor activities in Ghana that could influence salt intake between waves 2 and 3 of data collection. There are some risks in taking this pragmatic approach, although local experts will provide ongoing and active assessments in both countries between study waves, and this study team will undertake rigorous comparisons of the nested study groups at waves 2 and 3 to determine the feasibility of this research strategy.

### Study population

#### Random sampling procedure

WHO-SAGE is a multinational study examining the health and well-being of adult populations and the ageing process. Two waves of this longitudinal study have been completed in China, Ghana, India, Mexico, Russia and South Africa.^29^ Further details about WHO-SAGE can be found via the WHO website (http://www.who.int/healthinfo/sage/cohorts/en/) including access to data from SAGE wave 0 (2002–2004), SAGE wave 1 (2007–2010) and SAGE wave 2 (2014/2015) following the completion of data cleaning. SAGE wave 3 will be implemented in 2017, with fund-raising for SAGE wave 4 and beyond ongoing. Evaluation of the health effects of this sodium policy on South African adults, in comparison to Ghanaian adults, is conducted using a nested study design in waves 2 and 3, with monitoring of the nested cohort continuing in subsequent waves of data collection.

In total, 42 464 respondents were recruited across the six countries for wave 1 (2007–2010), including 4223 respondents in South Africa (9% 18–49 years; 40% 50–59 years; 51% 60+ years). Wave 1 respondents were recruited from selected probability sampled enumeration areas (EAs) using a multistage cluster sampling strategy, with stratification by province, residence and race.

The wave 2 sampling strategy was designed to account for expected attrition as a result of participants having moved house or died since wave 1, especially given that over half of the sample were already above 60 years of age in 2007. All wave 1 households were visited for wave 2 data collection (including a verbal autopsy for those participants no longer alive). Replacements for sample attrition used a systematic sampling approach to randomly select new households using EA aerial photographic maps on which dwellings are clearly visible, starting at a random point on the periphery of the EA and following predetermined routes. Households were then classified into the following mutually exclusive categories: (1) SAGE wave 1 follow-up households with one or more members aged 50 years or older targeted for selection; (2) new households with one or more members aged 50 years or older; (3) SAGE wave 1 follow-up households which include residents aged 18–49 targeted for selection or (4) new households which include residents aged 18–49. Younger adults are allowed to ‘age-in’ to the older adult group, with targeted refreshing of the youngest ages (18–23) in the younger adult sample.

The sampling method used in SAGE Ghana wave 2 followed a similar design, based on the 2003 World Health Survey/SAGE wave 0^30^ with primary sampling units (PSUs) stratified by region and location (urban/rural). Selection of the PSUs was based on proportional allocation by size using the same follow-up and random systematic sampling method as South Africa.

### Selection and data collection in the nested cohort

Both South Africa and Ghana include between 3500 to 4500 households for SAGE's main survey sample. The samples selected for urine collection (n=1200 in each country) from waves 2 and 3 of the main study are of adults aged 18+ years, with the final distribution in the main and nested studies reflecting the weighting towards recruiting more adults aged 50+ years. In South Africa, the nested study respondents were sampled from among the first wave 2 households visited within each probability sampled EA (day 1 in the EA). This approach was taken to prioritise the shipment of all collected urine samples to a central laboratory (Global Clinical and Viral Laboratory, Durban) within 3 days of collection while maintaining a cold chain regardless of where urine collection took place. This was necessary as there were no decentralised facilities available to freeze urine. The SAGE South Africa team used 20 survey teams (one nurse and three interviewers per team) simultaneously collecting data and urine/blood samples from respondents across all provinces in the country over a 5-month period (August to December 2015).

Selection of the nested study sample in Ghana differed slightly from the one in South Africa. All urine samples were collected by just four fieldwork teams (one research assistant and four interviewers per team) moving region to region over a 10-month period (September 2014 to June 2015). To facilitate this approach, EAs were randomly selected, with stratification by urban/rural, from the three geographical areas (savannah, forest and coastal) of Ghana and designated ‘urine EAs’ from which the target 1200 respondents were recruited.

Inclusion criteria for urine collection were: respondent must be part of the WHO-SAGE cohort, with no indication of urinary incontinence or other condition that could impede 24-hour urine collection; and if female, not menstruating, pregnant or breast feeding on the day of collection. All respondents who provided samples in wave 2 will be approached again in wave 3 in both countries, with procedures as described earlier for replacement and refreshment of the sample.

### Study measures included in WHO-SAGE

All survey teams are trained with support from WHO Geneva, with survey teams using standardised household, individual and proxy questionnaires, anthropometry, blood sampling, BP and physical function tests as described previously in SAGE wave 1.^29^ Study measures are listed in table 1 and translation and back-translation protocols are in place for the survey instruments. Interviewers speak the respondents' home languages with consent forms available in the most widely spoken languages for each area.

**Table 1:** Primary and secondary outcome measures

Variable	Primary/secondary outcome	Method of measurement
Sodium intake	Primary	24-hour urinary sodium excretion
Blood pressure	Primary	Validated, automated wrist BP monitor—triplicate measure
Potassium intake	Secondary	24-hour urinary potassium excretion
Iodine status	Secondary	Urine iodine concentration
Cigarette smoke exposure	Secondary	Urine cotinine concentration and questionnaire
Medication use	Secondary	Questionnaire
Dietary salt behaviour	Secondary	Questionnaire*
Alcohol consumption	Secondary	Questionnaire
Physical activity	Secondary	Global Physical Activity Questionnaire^44^
Body composition	Secondary	Height, weight, waist and hip circumference
Quality of life	Secondary	WHO Quality of Life instrument (WHOQoL)^45^

*The dietary salt questionnaire consists of the following questions: (1) Do you add salt to food at the table? (Always, rarely, sometimes, often, never); (2) In the food you eat at home, salt is added in cooking [always, rarely, sometimes, often, never); (3) How much salt do you think you consume? (Far too much, too much, just the right amount, too little, far too little, don't know, refused); (4) Do you think a high salt diet could cause a serious health problem? (Yes, no, don't know, refused); (5) Do you do anything on a regular basis to control your salt or sodium intake? (Yes, no, don't know, refused).

BP, blood pressure; WHOQoL, WHO Quality of Life instrument.

### Urine collection in the nested study (waves 2 and 3)

The study protocol used for sodium determination in 24-hour urine samples followed the WHO/Pan American Health Organization (PAHO) guidelines.^31^ Respondents were requested to collect all urine produced for 24 hours, excluding the first pass urine on day 1, but including the first urine of the following morning (day 2) in a 5 L plastic container using 1 g thymol as preservative. The spot sample was collected without preservative from the second urine passed on day 1 (marking the start of the 24-hour collection) and decanted into three 15 mL Porvair tubes (Porvair Sciences, Leatherhead, UK), then kept in a thermoelectric cooler box powered by the fieldwork vehicles and containing ice packs to maintain a lowered temperature. The 24-hour sample was collected the next morning, total volumes were recorded and aliquots (4×15 mL Porvair tubes) generated with all samples then shipped to the laboratory maintaining the cold chain using precooled ice packs as a means to maintain temperature control. When the samples arrived at the laboratory, the cooler box was examined and the temperature of the samples noted and recorded.

Owing to differences in available resources, duration of data collection and logistical challenges presented by each country, Ghana and South Africa used different methods of urine preservation in wave 2. In Ghana, the survey team kept urine samples in hospitals, clinics and health centres with laboratory facilities in the EA in which they were working. Samples were taken on ice to the central university laboratory between the first and fifth days of interviews in each EA. This was necessary as there were limited options and resources for commercial transport of samples within Ghana. Additionally, with Ghana's tropical climate and a smaller fieldwork team than in South Africa, the possibility that samples may stand for longer in a warmer climate prior to collection necessitated the use of 37% hydrochloric acid (HCl; AnalaR NORMAPUR) as a preservative added to both the 24-hour (10 mL per 5 L collection container) and spot urine (10 mL per 2 L collection container) samples.

In South Africa, thymol preservative, a crystalline natural derivative of the thyme plant, is used as it is easier and safer to transport and handle than commonly used liquid acids by the 20 operating fieldwork teams and courier companies, many of which will not transport liquid acids. In both countries, survey teams explain the dangers of the preservatives to respondents, and are trained in handling the preservatives and biological materials. Both thymol and HCl have been shown to prevent changes in urinary creatinine, sodium and potassium concentrations for up to 5 days.^32^

Incomplete 24-hour urine collections are assumed if: total volume ≤300 mL; or creatinine excretion ≤4 mmol/day (women) or ≤6 mmol/day (men).^33^ The use of para-amino benzoic acid (PABA) has been suggested as a more accurate method than creatinine to validate 24-hour urine collection completeness.^34^ However, PABA recovery rate declines with age in respondents older than 30 years.^35^ Considering this together with the increased risk for non-compliance and attrition due to the additional burden of remembering to take the PABA pill 3 days before the urine collection, as discussed in the WHO/PAHO guidelines for sodium determination in 24-hour urine samples,^31^ PABA is not used in this study.

### Urine analysis

Sodium and potassium were determined using the indirect ion-selective electrode method and creatinine analysed using the standardised urinary Jaffe kinetic method (South Africa: Beckman Coulter Synchron DXC600/800 System; Ghana: BioSystems Analyzer A25). The WHO population target for salt intake is 5 g salt (NaCl) per day, equivalent to urinary sodium excretion 85 mmol/24 hour. Urinary potassium should be >70 mmol/24 hour, with a sodium-to-potassium ratio <1 shown to be protective for all-cause, cardiovascular and ischaemic heart disease mortality.^15^ With the exception of iodine, all South African samples were analysed at a single laboratory in Durban (Global Clinical and Viral Laboratory). In Ghana, with the exception of iodine, all samples were analysed at a single laboratory in Accra (University of Ghana Chemical Pathology Laboratory in the School of Allied Health Sciences).

Urine samples for iodine analysis from both countries were stored at −20° C and batch analysed using the Sandell-Kolthoff method with ammonium persulfate digestion and microplate reading^36^ at the North-West University Centre of Excellence for Nutrition. The laboratory participates successfully in the Program to Ensure the Quality of Urinary Iodine Procedures (EQUIP, US Centres for Disease Control and Prevention, Atlanta, Georgia, USA).^37^ A median of <100 μg iodine/L indicates population-level deficiency (there is no reference range for individuals).^38^

Even though there is no evidence that the addition of preserving substances such as HCl and thymol affects urinary iodine concentrations,^39^ we undertook testing to examine the influence of adding thymol or HCl to urine samples (n=20) on urinary iodine concentrations. The results indicated no significant or relevant (below assay coefficient of variation) differences when compared with samples without added preservatives (results not shown here).

### Comparison of spot and 24-hour urine analyses

The following equations will be used to assess the accuracy of using spot urine samples to assess 24-hour urinary sodium, potassium and iodine excretion: (1) Tanaka;^40^ (2) Kawasaki^41^ and (3) INTERSALT.^42^ The sensitivity of the formulae to estimate the measured 24-hour urinary creatinine, sodium and potassium excretion will be assessed using the receiver operator characteristic curve analysis. Multiple regression modelling will be conducted to determine whether new regression models are more appropriate for use in African populations.

### BP measurements

Wrist-worn Omron BP devices were used to record three sequential measures on the left arm (1 min between each measure), with the wrist resting at the level of the heart and the respondent seated with legs uncrossed. The wrist BP devices are validated to the European Hypertension Society International Protocol.^43^

### Data capture, analysis and statistical power

All data were and will be captured using an electronic data capture system and uploaded to a secure central server within each country. Cleaning and analysis of survey data are coordinated by the WHO. Based on the modelled estimated reduction of 0.85 g salt per day as a result of the legislation, with a current estimated South African population salt intake of 8.1 g/day^12^ and a population variance of up to 35 g/day based on previous pilot data (unpublished), a sample size of 761 respondents would give 80% power to detect a significant difference (95% CI). Allowing for error in 24-hour sample collection (incomplete or missing samples) in this complex field study, a target sample size of 1200 was chosen.

### 

## Ethics and dissemination

All respondents provide written informed consent prior to taking part in the study. The study complies with the ethical principles for medical research involving human participants as per the Declaration of Helsinki.^46^ Baseline data collection (for this nested substudy as part of wave 2) is complete and the results will be published in peer-reviewed international journals, presented at national and international conferences, and summarised in research and policy briefs. Completion of the first follow-up of the nested study (as part of SAGE wave 3) in South Africa and Ghana is expected by December 2017. Dissemination of the final results will begin soon thereafter. All de-identified data will be made available in the public domain.

## Discussion

The WHO recommends that adults consume no more than 5 g of salt per day^47^ while most South African adults consume well above this level, with bread being the single highest contributor to non-discretionary sodium intake.^7^ In the light of the high prevalence of hypertension within the country,^3^ the government's bold move to reduce sodium in bread, cereals, snacks, processed meats, spreads, soups, stocks and gravy in 2016, with further reductions mandated for 2019, is welcomed. This intervention could significantly reduce the prevalence of hypertension, stroke and CVD in South Africa, thereby substantially reducing healthcare costs to the state and to individuals.^12^ ^13^ However, successful reduction of population level sodium intake through legislation has yet to be demonstrated.

The need to simultaneously monitor both sodium reduction and iodine status is required as, in South Africa, universal salt iodisation has successfully eradicated iodine deficiency.^48^ ^49^ The two public health strategies are compatible^50^ if ongoing surveillance informs the adjustment of iodine levels in iodised salt as population salt intake decreases.^51^ The assessment of salt behaviours will be interesting, as multiple strategies accompany the sodium legislation, including mass media campaigns such as SaltWatch, which is coordinated by the Heart and Stroke Foundation South Africa,^52^ and primary care health education activities conducted by the South African (NDoH). All SAGE countries will include the standard salt behaviour questions in waves 2 and 3 so that a cross-national comparison of salt behaviours will be possible between the six LMIC countries.

### Assumptions and risks in the causal logic

We make several assumptions within the implementation portion of the Results Chain model (figure 1) including the timely compliance of all manufacturers of the targeted food products with the sodium legislation; and that educational activities reach the intended beneficiaries.

South Africa is now one of a number of countries with mandatory sodium targets; others include Argentina, Belgium, Bulgaria, Greece, Hungary, the Netherlands, Paraguay and Portugal.^6^ ^18^ Mandatory sodium targets in Argentina (Act 26905) came into force in December 2014 for three food groups: (1) meat and meat products; (2) soups, dressings and canned foods; and (3) farinaceous or starch containing products such as crackers, cookies and bakery products.^53^ There are strict penalties for not meeting the sodium regulations, ranging from fines to the confiscation of food products, and suspension of business for up to 5 years.^53^ A prepolicy evaluation conducted in February 2014 comparing food labels against the mandatory targets concluded that most foods in Argentina were already meeting the target sodium levels, though this differed by food category with around half of the bakery products reviewed exceeding the mandated sodium level.^54^ This high prelegislation compliance may be explained by the successful government-led programme ‘Less salt, more life’ initiated in Argentina in 2011, including voluntary agreements with the food industry to lower sodium in foods and extensive monitoring of both implementation (food sodium testing in independent laboratories) and effectiveness (sodium intake surveys and urinary sodium measurement).^55^ In contrast to this example is Belgium, where mandatory sodium levels in bread were set in 1985, although in 2006 half of the bakers were still producing bread with higher sodium levels, stating that tighter regulation led to products being imported and unfair competition from neighbouring European countries.^56^ Only after an awareness campaign was directed specifically at bakers did compliance increase to 90% by 2008, 13 years after the legislation was introduced.

Findings from Argentina also highlight that sodium discussions often take place between government and the bigger food companies and an ongoing challenge will be to determine compliance with sodium targets in small-sized and medium-sized food producers.^54^

These examples highlight the need for continued compliance monitoring, an understanding of the reasons for non-compliance, and clear interventions and penalties for non-compliance, as it would seem that compliance with legislation cannot be taken for granted.

Non-compliance by targeted food manufacturers or increased salt levels in non-legislated food products (by other food producers) are risks to undermining the intended outcomes. Educational activities that do not reach the intended beneficiaries are also a risk as they may fail in the message to modify discretionary salt use. Media campaigns to reduce salt can work,^57^ and the key again will be in documenting and monitoring their success. As a mitigation strategy for these risks, the SAGE South Africa team is working closely with stakeholders in government, academia, non-governmental organisations and research organisations who are directly involved in monitoring compliance and/or the development, delivery and evaluation of salt and BP educational activities. In September 2016, stakeholders met to develop a roadmap for the South African salt reduction strategy, with action points and a report due to be published. The meeting serves as a foundation to coordinate and link efforts. The authors will continue discussions with these stakeholders and are open to any discussions that promote a thorough and valid evaluation of the effectiveness of this important health policy.

Also, within the Results Chain model (figure 1), several assumptions underlie achieving the results including consumers purchase and consume the lower sodium products; educational activities are effective in changing behaviour to decreasing discretionary salt use; reduced salt intake is accurately reflected in a single 24-hour urine collection; reduced salt intake leads to reduced BP and ultimately a reduction in CVD events and healthcare spending. There is strong evidence supporting the final outcomes with high salt intakes associated with increased risk of stroke and CVD^58^ ^59^ and population efforts to lower sodium intake producing notable improvements in BP and deaths from stroke and CVD.^60^ Naturally, this does not guarantee that we will find the same outcome. WHO-SAGE is well placed to continue follow-up in the South African cohort, as it has been operational in South Africa since 2003 with wave 0 of data collection,^29^ and plans to continue data collection after wave 3 (2017) approximately every 4 years. This provides an ideal opportunity to collect longer term data on cardiovascular and stroke mortality and morbidity in South Africa and Ghana.

In terms of acceptability of the lower sodium foods, one systematic review suggests that sodium levels can be reduced by up to 40% in bread and 70% in meat products without compromising consumer acceptability.^61^ Additionally, longer term exposure to lower salt foods has been shown to increase preference for those foods and decrease preference of higher salt alternatives.^62^ ^63^ While it would appear that sodium regulations are unlikely to modify consumer purchasing behaviour, the WHO-SAGE South Africa team is also collaborating with organisations able to monitor consumer food purchasing patterns prelegislation and postlegislation.

There is evidence supporting salt reduction campaigns change behaviour and impact salt intake. For example, a community-based health education programme in Japan successfully decreased salt intake by 2–3 g/day.^57^ A campaign in the UK decreased discretionary salt use^64^ and reduced overall salt intake by around 10%.^65^ Again, monitoring and evaluation of educational activities within South Africa will be important to determine their effectiveness.

Finally, there is the question of whether one 24-hour urine collection is sufficient to assess dietary salt intake. There is little doubt that repeated 24-hour urine collection in an individual improves the accuracy to assess salt intake, with more collections giving greater accuracy.^66^ However, repeated sampling may lead to refusals or more incomplete samples^67^ producing underestimates of salt intake.^68^ Additionally, Mente *et al*^67^ point out that large studies reduce random error, so that average estimates of salt intake from 24-hour urine collection across a large number of people are reliable estimates for groups, albeit less so for individuals. While repeated 24-hour urine collection may be feasible in smaller randomised controlled trials, it can be impractical for larger studies, especially in LMICs.^69^ Furthermore, 24-hour urine collection remains the gold standard to measure salt intake when compared with estimated intake from casual or spot urine samples.^70^ Therefore, a single, well-collected 24-hour urine sample appears a pragmatic approach to take in this prospective cohort study of over 1000 participants in each of the two countries.

### Limitations of the study

WHO SAGE is designed to investigate the health and ageing process in older ages; therefore, the focus is on adults aged 50 years and older. Although a smaller sample of adults aged 18–49 years is included, the results from this study may not be representative for younger adult age groups. However, SAGE wave 1 data showed that four in every five adults above 50 years of age in South Africa are hypertensive,^3^ justifying a focus on monitoring the effectiveness of the sodium legislation within this age group. The gold standard method for estimating sodium intake is used (24-hour urinary Na excretion), yet the lack of comprehensive dietary assessment will not allow identification of major food contributors to total salt intake. Another limitation is our ability to effectively monitor the food industry adherence to the legislation. While we will work with key stakeholders to jointly evaluate the degree of enforcement observed and sodium content of food products, this is not the primary aim of the study. One potential limitation could be the choice of comparison group. Using Ghana provides a reasonable choice with some similar demographic and epidemiological characteristics and without formal national-level sodium legislation. While standard training and interview techniques as well as survey instruments were used, there was a difference of several months in the time frame for wave 2 data collections in South Africa and Ghana. It is unclear if this will impact the validity of comparisons. A clear analytical plan will be in place to facilitate interpretation of results by WHO-SAGE, SAGE-Ghana and SAGE-South Africa teams.

### Strengths of the study

The comprehensive assessment of the ageing process within SAGE, alongside the rigorous random selection procedures employed, provides an ideal opportunity to assess the effectiveness of this legislative public health approach. Additionally, WHO SAGE is ideally placed for ongoing data collection to include evaluation of the planned 2019 legislative changes to further reduce sodium levels in foods and collection of data on hard end points such as cardiovascular-related mortality which require a longer time frame. WHO-SAGE measures BP in each wave of data collection and will continue to do this. While urinary sodium analysis is conducted only for waves 2 and 3 in the nested studies in South Africa and Ghana, from wave 2 onwards, questions on discretionary salt use have been added to SAGE for all respondents in each of the six countries. This will provide valuable data on self-reported discretionary salt behaviours as countries implement various strategies to reduce population salt intake.

Considering the burden of potentially preventable hypertension on health services in South Africa, the government's population-level advance towards making the food supply healthier is innovative. The current study adopts a novel approach to evaluate this public health policy in a cost-efficient and pragmatic manner, generating information relevant to other countries, as sodium legislation is increasingly being adopted by governments across the globe.
